# Deep-learning approach for automated thickness measurement of epithelial tissue and scab using optical coherence tomography

**DOI:** 10.1117/1.JBO.27.1.015002

**Published:** 2022-01-18

**Authors:** Yubo Ji, Shufan Yang, Kanheng Zhou, Holly R. Rocliffe, Antonella Pellicoro, Jenna L. Cash, Ruikang Wang, Chunhui Li, Zhihong Huang

**Affiliations:** a University of Dundee, School of Science and Engineering, Dundee, United Kingdom; b Edinburgh Napier University, School of Computing, Edinburgh, United Kingdom; c University of Glasgow, Center of Medical and Industrial Ultrasonics, Glasgow, United Kingdom; d The University of Edinburgh, The Queen’s Medical Research Institute, MRC Centre for Inflammation Research, Edinburgh, United Kingdom; e University of Washington, Department of Bioengineering, Seattle, Washington, United States

**Keywords:** optical coherence tomography, deep-learning network, wound healing, re-epithelialization, epidermis, scab

## Abstract

**Significance:**

In order to elucidate therapeutic treatment to accelerate wound healing, it is crucial to understand the process underlying skin wound healing, especially re-epithelialization. Epidermis and scab detection is of importance in the wound healing process as their thickness is a vital indicator to judge whether the re-epithelialization process is normal or not. Since optical coherence tomography (OCT) is a real-time and non-invasive imaging technique that can perform a cross-sectional evaluation of tissue microstructure, it is an ideal imaging modality to monitor the thickness change of epidermal and scab tissues during wound healing processes in micron-level resolution. Traditional segmentation on epidermal and scab regions was performed manually, which is time-consuming and impractical in real time.

**Aim:**

We aim to develop a deep-learning-based skin layer segmentation method for automated quantitative assessment of the thickness of *in vivo* epidermis and scab tissues during a time course of healing within a rodent model.

**Approach:**

Five convolution neural networks were trained using manually labeled epidermis and scab regions segmentation from 1000 OCT B-scan images (assisted by its corresponding angiographic information). The segmentation performance of five segmentation architectures was compared qualitatively and quantitatively for validation set.

**Results:**

Our results show higher accuracy and higher speed of the calculated thickness compared with human experts. The U-Net architecture represents a better performance than other deep neural network architectures with 0.894 at F1-score, 0.875 at mean intersection over union, 0.933 at Dice similarity coefficient, and 18.28  μm at an average symmetric surface distance. Furthermore, our algorithm is able to provide abundant quantitative parameters of the wound based on its corresponding thickness maps in different healing phases. Among them, normalized epidermal thickness is recommended as an essential hallmark to describe the re-epithelialization process of the rodent model.

**Conclusions:**

The automatic segmentation and thickness measurements within different phases of wound healing data demonstrates that our pipeline provides a robust, quantitative, and accurate method for serving as a standard model for further research into effect of external pharmacological and physical factors.

## 1 Introduction

The skin is the largest organ of the human body and provides essential functions to maintain homeostasis of the body. One of the most important roles of the skin is to protect the body against harmful pathogens which exist in the external environment. Any form of injury initiates a rapid response to restore the integrity of the skin and remove potential invading pathogens. The process of tissue repair following injury is a fundamental process of all living organisms and can even be observed in primitive multi-cellular organisms. Despite species and tissue specificity to the tissue repair process, there are four canonical overlapping phases involved in the wound healing process: hemostasis, inflammation, proliferation, and remodeling.^1^

Impaired wound healing can be life-threatening,^2^^,^^3^ especially for sufferers of diabetes mellitus who can be at an elevated risk of developing chronic, non-healing wounds.^4^ Re-epithelialization is a critical procedure of wound healing that occurs during the proliferation and migration phases of wound healing. In short, all the wounds are covered by an epithelium (as an obstacle) administered by several complex events emanating from the epithelium itself and by the temporal recruitment into the wound bed for immune cells.^5^^,^^6^ Inability to re-epithelialize is a hallmark of chronic non-healing wounds.^7^ Note that the epidermis detection, such as the epidermal thickness, is an essential indicator to judge whether the re-epithelialization process is normal.^8^ Formation of a scab is also an essential indicator, commonly formed in the coagulation and inflammation phases, to provide structural stability to the wound and prevent exsanguination.^9^ Thus, to design effective treatments, further precise analysis of epidermal restoration and scar formation/loss during wound healing is required.

The complexity of the re-epithelialization process in wound healing cannot currently be replicated *in vitro*. The use of rodent model is an effective way of studying this process.^10^ Histology remains the gold standard for assessing the molecular and cellular change of rodent model quantitatively during wound healing.^11^ Non-invasive methods are, however, desirable because it eliminates the need either to sacrifice animals or to collect serial skin biopsies to evaluate changes in wound cure. Additionally, it may provide immediate information without changing the tissue conditions during imaging. Currently, optical coherence tomography (OCT) and OCT angiography (OCTA) are emerging three-dimensional (3D) and non-traumatic imaging modalities that are enable providing high-resolution volumetric tissue structural and vascular information up to a few millimeters in depth without contrast agent.^12^^,^^13^ With the advances in optical fiber and laser technology, OCT is also well-suited to investigate tissue responses in real time in high scattering tissue, especially in skin sites.^14^^,^^15^ In some studies, OCT and OCTA techniques have been explored to visualize microstructure and microvascular change in the process of wound healing in human and animal models.^16^^–^^19^

Quantitative analysis of epidermis and scab region based on OCT often includes manual segmentation, which is extremely time-consuming and impractical. Recently, researchers in the OCT community developed semiautomated epidermis segmentation methods to tackle this problem.^20^^–^^22^ The most common one utilized a number of user-defined lines located at the boundaries between different layers or features in 3D OCT volumes for segmentation.^21^^,^^23^^,^^24^ For the automatic segmentation and thickness quantification, Weissman et al.^25^ used a shapelet-based image processing technique. Hori et al.^26^ suggested to automatically detect the dermal-epidemic junction (DEJ) based on minimum local intensity. Li et al.^27^ defined the epidermis segmentation in three stages: low-square weighed-preprocessing, graphic surface detection of the surface of the skin, and local integral DEJ detection projections. However, segmentation process proposed in these studies is highly reliant on the image quality. It is prone to the segmentation errors if large variances of skin pathologies based on OCT are present. Srivastava et al.^28^ proposed a 3D graph-based approach to segment skin layers with a new cost function which is capable of degrading the impact of shadowing effects in OCT images. In some cases, however, the segmentation performance was significantly degraded when hair was touched the skin surface in B-scans.

To address this problem, a combination of machine learning (random forests and kernel regression) and mathematical modeling was investigated to support the graph-based segmentation.^29^^,^^30^ A combination of U-Net model and a robust postprocessing method was proposed to segment epidermis and hair follicle in healthy human skin.^31^ Kepp et al.^32^ developed a modified convolutional neural network (CNN) model by densely connected convolutions blocks rather than standard convolution blocks to segment the skin layer of healthy mouse. However, the epidermis and dermis layer were treated as one class in this study, which was impossible to obtain epidermis layer thickness information. Sheet et al.^33^ subsequently used self-induced denoising encoders to learn tissue-specific presentations. However, the standard quantitative evaluation for each phase of wound healing was missing. Moreover, no scab region was mentioned in their cutaneous wound healing model. Hence, the authors possibly treated the scab and epidermis region as one class.

Despite recent work on analyzing the processes of cutaneous wound healing and layer thickness computation using a mouse model, the applications based on OCT remain limited. Additionally, lack of a standardized segmentation method and quantitative analysis strategy based on wound healing model makes it difficult for researchers to gain access to the characteristics of epidermal and scab changes precisely during each phase of wound healing.

In this paper, we propose a pipeline based on deep-learning-based methods to segment the epidermis and scab region automatically in the process of wound healing (day 3, day 7, day 10, day 14 after injured, and control data). After training five deep-learning architectures and 10-fold cross evaluation, the segmentation performance of the five models was compared qualitatively and quantitatively. U-Net model^34^ has shown the best performance in segmenting the target structure while minimizing the effect of other low scattering interfering tissue and noise. This model also has higher accuracy of segmentation with higher F1-score compared with other models. Furthermore, our novel pipeline is able to provide the standardized thickness measurement of epidermis and scab inner and outer of the wound from *en face* reconstructed thickness maps.

Four contributions have been made related to proposed pipeline. (1) Various structural characteristics associated with the multiple phases of full-thickness excisional wound healing are successfully monitored in non-invasive way using standardized mouse model. (2) Our proposed pipeline is robust and efficient to segment target structures avoiding artifacts that are caused by many image acquisition processes [hyper-reflection, bulk noise, and signal-to-noise ratio (SNR) gapping] and diverse anatomy (remaining mouse hair, epithelial tongue, and granulate tissue). Importantly, our approach has the ability to distinguish the scab and epidermis region. (3) A robustly accurate measurement method is proposed even when neither the skin surface nor the dermoepidermal junction is flat in the wound region during various wound healing stages. (4) A new way of measuring normalized epidermal thickness is provided to quantitatively measure the re-epithelialization process in the wound area over the healing timeline without manual intervention. This lays the groundwork for rapid clinical quantitative translation, which will improve the existing wound healing evaluation technique in mice.

## 2 Method

### 2.1 Deep-Learning-Based OCT Pipeline for Automatic Measurement of Epidermis Layer and Scab

**Fig. 1:**
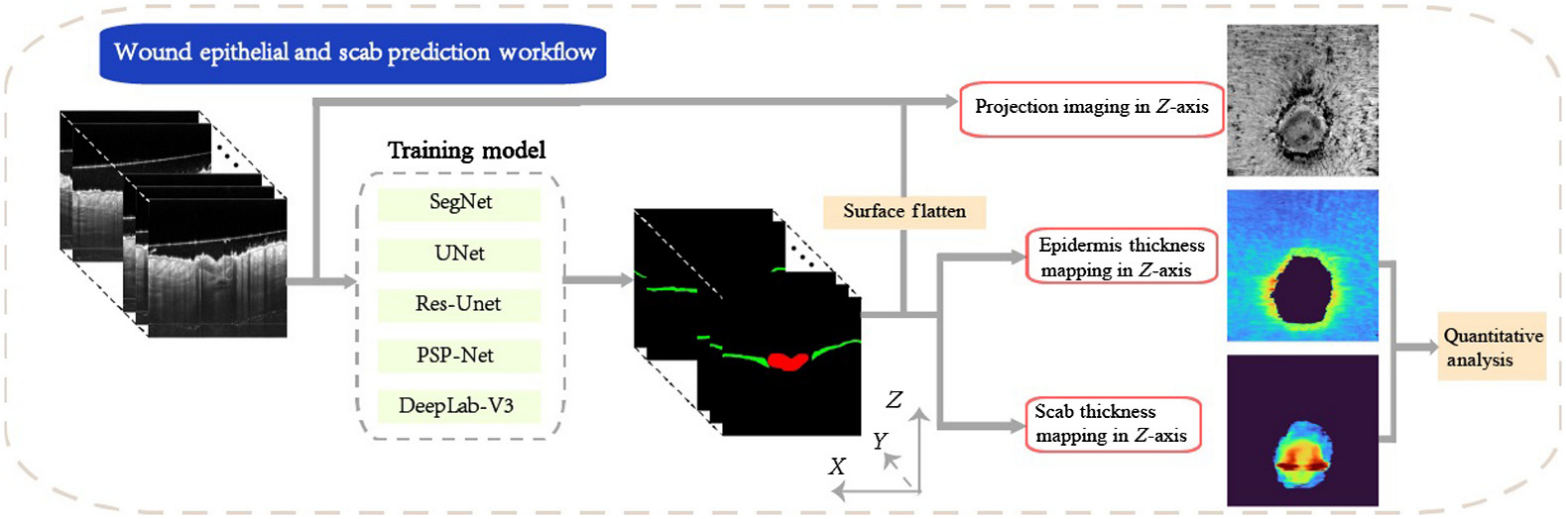
The pipeline of automatic measurement of epidermis layer and scab. Five network structures of deep-learning network are compared in this study (Seg-Net,^35^ U-Net,^34^ Res-UNet,^36^ PSP-Net,^37^ and DeepLab-V3^38^).

To facilitate the use of OCT for monitoring wound healing, an automatic algorithm to quantify the thickness of the epidermis and scab is desired. We deployed deep-learning methods for the segmentation of the epidermis and scab from each cross-section OCT images during wound healing and then created a standardized strategy to automatically evaluate layer thickness from reconstructed *en face* thickness maps. Figure 1 provides a schematic description of the proposed pipeline. Our proposed pipeline comprises the following three steps:

1. Generation of a structural image using previously developed reconstruction method;
2. Automatic segmentation of the epidermis and scab layers from cross-section view using CNN-based deep-learning algorithms;
3. Evaluation of the thickness of epidermis and scab layer using automatic algorithms based on *en face* thickness maps and proposed calculation strategy.

### 2.2 Experimental Setup

#### 2.2.1 Experimental samples

All experiments were conducted with approval from the local ethical review committee at the University of Edinburgh and in accordance with the UK Home Office regulations (Guidance on the Operation of Animals, Scientific Procedures Act, 1986). Experiments on animals were performed under PIL I61689163 and PPL PD3147DAB (January 2018 to January 2021). Experiments were performed on 8-week-old male (8W) C57Bl/6J wild-type mice (Charles River Laboratories, Tranent, UK).

Two mice that underwent the OCT examination were anesthetized with isoflurane (Zoetis, Leatherhead, UK) by inhalation. Prior to wounding, animals received a subcutaneous injection of analgesia (buprenorphine 0.05  mg/kg) (Vetergesic, Ceva Animal Health Ltd., Amsterdam) and the hair was trimmed on the dorsal skin (BaByliss Super Motor Skeleton Trimmer; BaByliss, Hampshire, UK). Remaining fur was depilated with Nair sensitive hair removal cream (Church and Dwight, Folkstone, UK). Four full-thickness excisional wounds were made to the dorsal skin using a sterile, single-use, 4-mm-punch biopsy tool (Kai Medical; Selles Medical, Hull, UK). Mice were housed in conventional cages in a 28°C warm box (Scanbur, Denmark) overnight following the wounding.

#### 2.2.2 System setup and imaging protocol

**Fig. 2:**
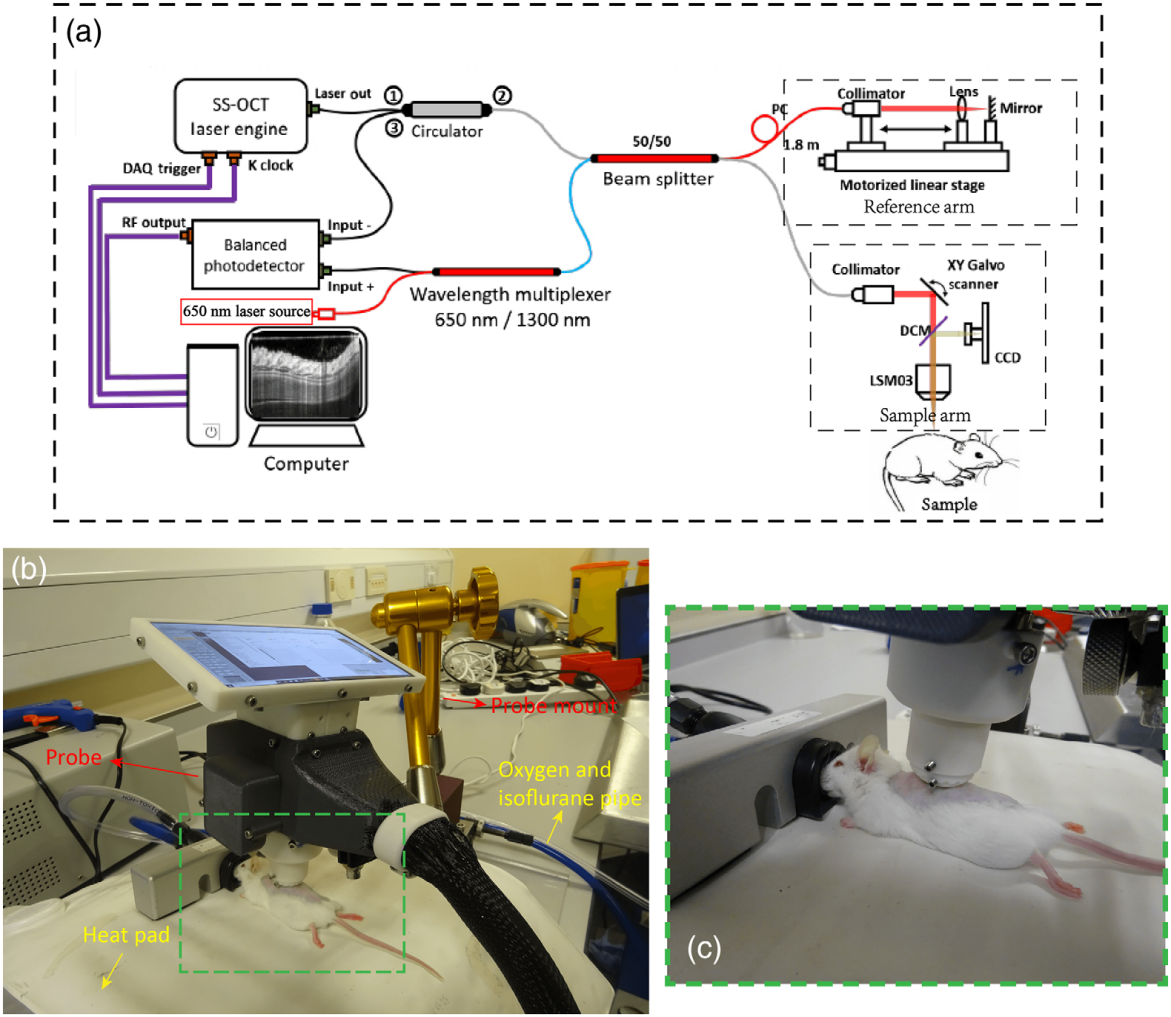
Experimental prototype of OCTA system. (a) Experimental setup based on SS-OCTA system. (b) Photo showing one mouse being imaging using the proposed experimental setup. (c) A magnified view of the area denoted by green dashed rectangle in (b).

The system used for this study was an in-house-built, experimental prototype swept source-OCT (SS-OCT) system, as shown in Fig. 2(a). This SS-OCT system was illuminated by a 200-kHz vertical-cavity surface-emitting swept laser source (SL1310V1-20048, Thorlabs Inc., Newton, NJ, USA). The light source has a central wavelength of 1310 nm and a spectral bandwidth of 100 nm, giving an axial resolution of ∼8  μm in tissue (∼11  μm in air). The sample arm consisted of a hand-held probe, where a pair 2D galvo-scanner, an objective lens (LSM03, Thorlabs Inc.), collimator, and display system (a mini charge-coupled device camera and a mounted screen) were housed. The probe was affixed with a sample space to maintain a consistent distance between the objective lens and the mouse skin. To minimize the bulk motion caused by breathing of mouse, a 5-mm thickness and 15-mm diameter round cover glass was used. Between the mirror and the skin, ultrasound gel was applied in the region of interest (ROI) which could reduce the specular reflections from the superficial layer of skin.^39^ Moreover, the gel could fill the uneven surface around the mouse wound to further reduce the effect from mouse breathing. A visible laser beam with a wavelength of ∼650  nm was also including in the system, which was used to guide scanning area basically in the center of the injured region. 3D scanning captured by this SS-OCT system contained 400×400  pixels, providing a field of view of $4\times 4\;{\mathrm{mm}}^{2}$. Four repeated B-scans were taken at the same position of B-scan to extract the blood flow from static tissue. The moderate penetration depth can be obtained for ∼1  mm. The scanning time for each 3D volume was ∼6  s, and each wound was repeatedly scanned for 3 to 5 times.

The imaging procedure is shown in Fig. 2(b), and the magnified images are showed in Fig. 2(c). Mouse body temperature was maintained at 37°C with a heating mat. Scanning session for each mouse, including preparation and adjustment of optimal position to ensure the adequate stability of the probe during the imaging, was <25  min for animal safety purpose. With the setups, three out of four wounds in the dorsal skin for each mouse were scanned successfully. One extra scanning was also taken on healthy skin adjacent to the wound for comparison purposes.

### 2.3 Data Processing

**Fig. 3:**
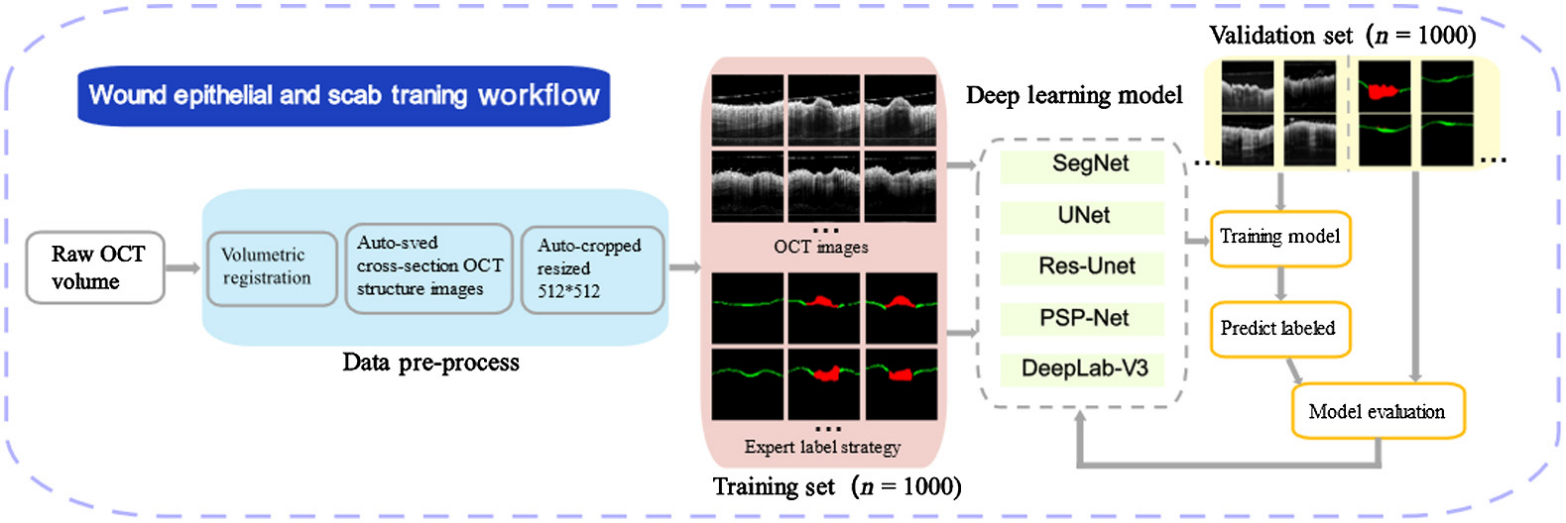
The pipeline of training workflow for automatic measurement of epidermis layer and scab.

Each acquired OCT and OCTA volume with size of 400×400×1920 (length × width × depth in pixels) were preprocessed first. Afterward, the processed images were used to train deep-learning neural networks. Figure 3 shows schematic description of the training procedure of the semantic work. The detailed procedure is outlined step-by-step in this section.

#### 2.3.1 Preprocess

The preprocessing of the acquired raw data volumes was performed using customized MATLAB scripts (MATLAB 2020a, MathWorks Inc, Natick, MA, USA). OCT cross-sectional structural image was attained by forming the average of the repeated OCT signals at the coequal spatial position.

To decrease the inevitable mice motion artifact and speckle noise, we used an Elastix-based 3D registration method that included rigid affinity and non-rigid B-spline transformation repeated volume registration and averaging.^40^^,^^41^ Inter-B-frame complex eigendecomposition-OCTA algorithm was applied onto the repeated B-frames at each position to extract microvascular network information.^42^

Each B-scan was first cut to remove unnecessary background and then resized to 512 to 512 for greater computational efficiency. To reduce computational time, other preprocessed methods, such as, contrast enhanced, attenuation compensation, or shadow removal algorithm have not been applied for collection of cross-section structure images.

#### 2.3.2 Database

All images were digitally stored in float 16 data format for offline analysis. Due to strong motion and shadow artifacts, four volumes had to be excluded, resulting in a total number of 36 OCT volumes, consisting of 14,400 OCT cross-section images in total (36 volumes × 400 B-scans/each volume). One thousand full-size 2D B-scan images from mouse No. 494 were randomly selected (200 for each phase) for training datasets, and 1000 randomly selected 2D B-scan images from mouse No. 505 were used as a validation set. The model selection was based on the quantitative analysis of validation datasets. In total, 2000 B-scan images were annotated by two experts using a custom software by MATLAB.

#### 2.3.3 Deep neural network training

It is a difficult task to process cross-sectional OCT images with hyper-reflection, bulk motion, and SNR gapping. Thus, finding proper architecture is an essential step in our study. In our previous study,^43^ dense fully convolutional network and full resolution convolution network always perform over-or under-segmentation especially with low-contrast medical images. Furthermore, an executive review that we have done through previous researchers^43^^–^^45^ has also been confirmed with previous studies. The selected five architectures cover three types of structures: Seg-Net, U-net, and Res-UNet, which have all adopted the symmetric structures for encoder and decoder; PSP-Net belongs to U-Net variation. Unlike U-Net, PSP-Net captures multi-scale spatial context from deep layers; DeepLab-V3 belongs to Res-UNet variation. Unlike Res-UNet, it integrates dilated convolution and spatial pyramid pooling into the architecture.

**Fig. 4:**
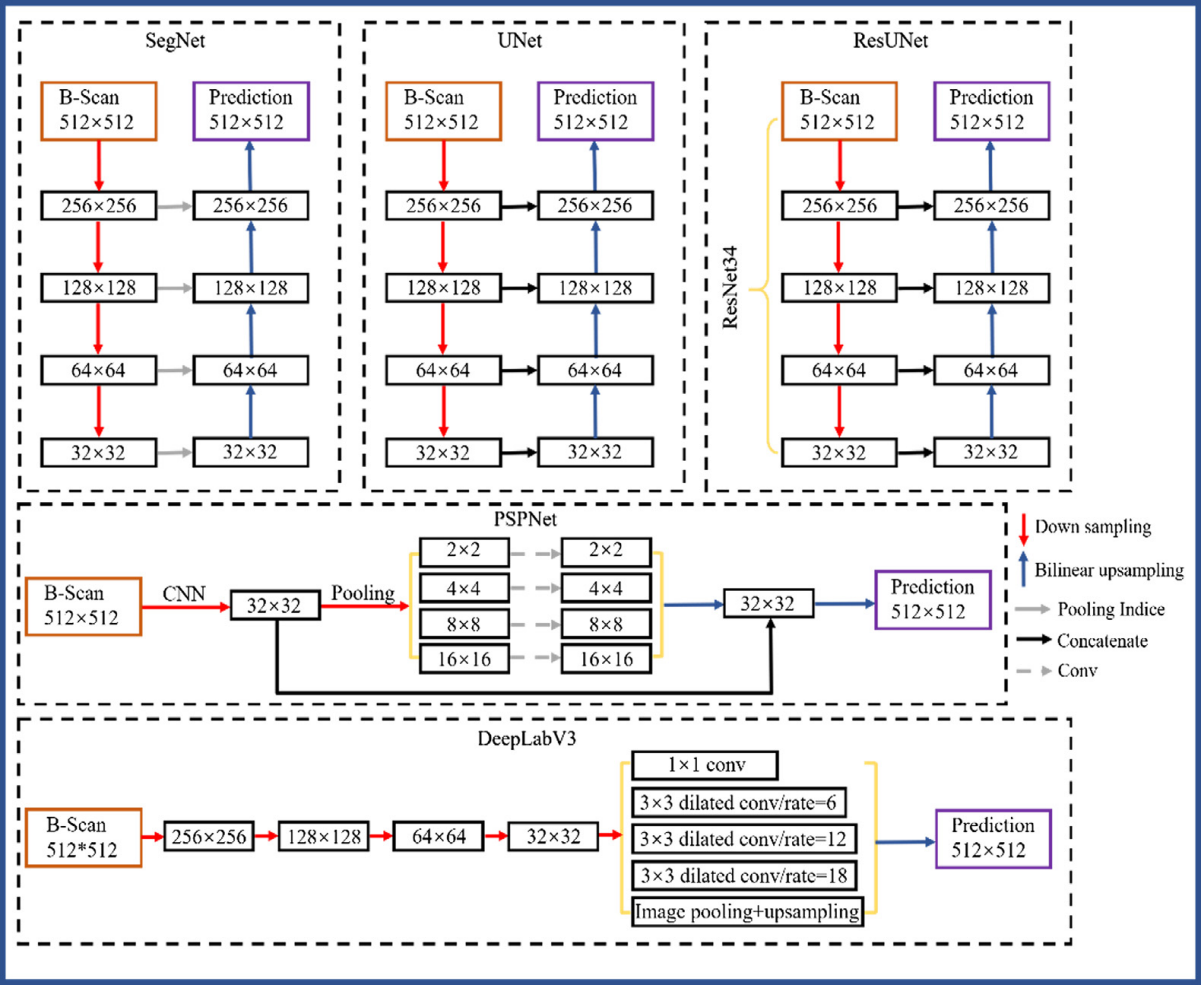
Architecture of five different CNN-based models (Seg-Net, U-Net, Res-UNet, PSP-Net, and DeepLab-V3).

The five CNN-based architecture can be observed in Fig. 4 to address the segmentation task. Data augmentation is performed to prevent overfitting. We randomly applied horizontal flips and rotations (±15  deg) to each input B-scan image. The data were augmented using horizontal flips (left to right/right to left). For each epoch, each image was randomly flipped horizontally with a 50% chance. No early stoppage is used, with convergence based on validation loss inspections. Each network is trained from scratch with weights initialized with utilizing the normalization approach of Glorot and Bengio.^46^

In the study, the Adam algorithm was used for minimizing the sum of cross entropy losses. The hyperparameters used in Adam are α=0.001, β1=0.9, β2=0.999, $\epsilon =1\times 10^{-8}$. The cross-entropy loss function is defined as

$$L_{i}=-y\;\log(P_{i})-(1-y_{i})\log(1-p_{i}),$$ (1)

where $y_{i}$ is the targets and $p_{i}$ represents the class probabilities. The sigmoidal outputs of a neural network can be defined as follows:

$$\sigma (z_{i})=\frac{1}{1+e^{-z_{i}}}.$$ (2)

For fair comparison, the strategy of data augmentation, initialization, and training parameters remain to the same for five CNN-based architectures. The specific training parameters are shown in Table 1. The software environment used throughout this work consists of Keras 2.2.446 using Tensorflow47 (GPU) 1.8.0 backend in Python 3.7.10. The hardware consists of an Intel Xeon^®^ 3.30 GHz E5-2680 v3 CPU, Nvidia GeForce GTX 1080Ti GPU, VMware virtual SSD and 16 GB 2400 MHz DDR4 ECC RAM.

**Table 1:** Training parameters for five CNN-based models.

Training parameters	Value
Batch size	8
Epoch	200
Learning rate	0.0001
Weight decay	$2\times 10^{-5}$
Optimizer momentum parameters	0.9
Decay of learning rate	0.99

### 2.4 *En Face* Thickness Measurement Method

**Fig. 5:**
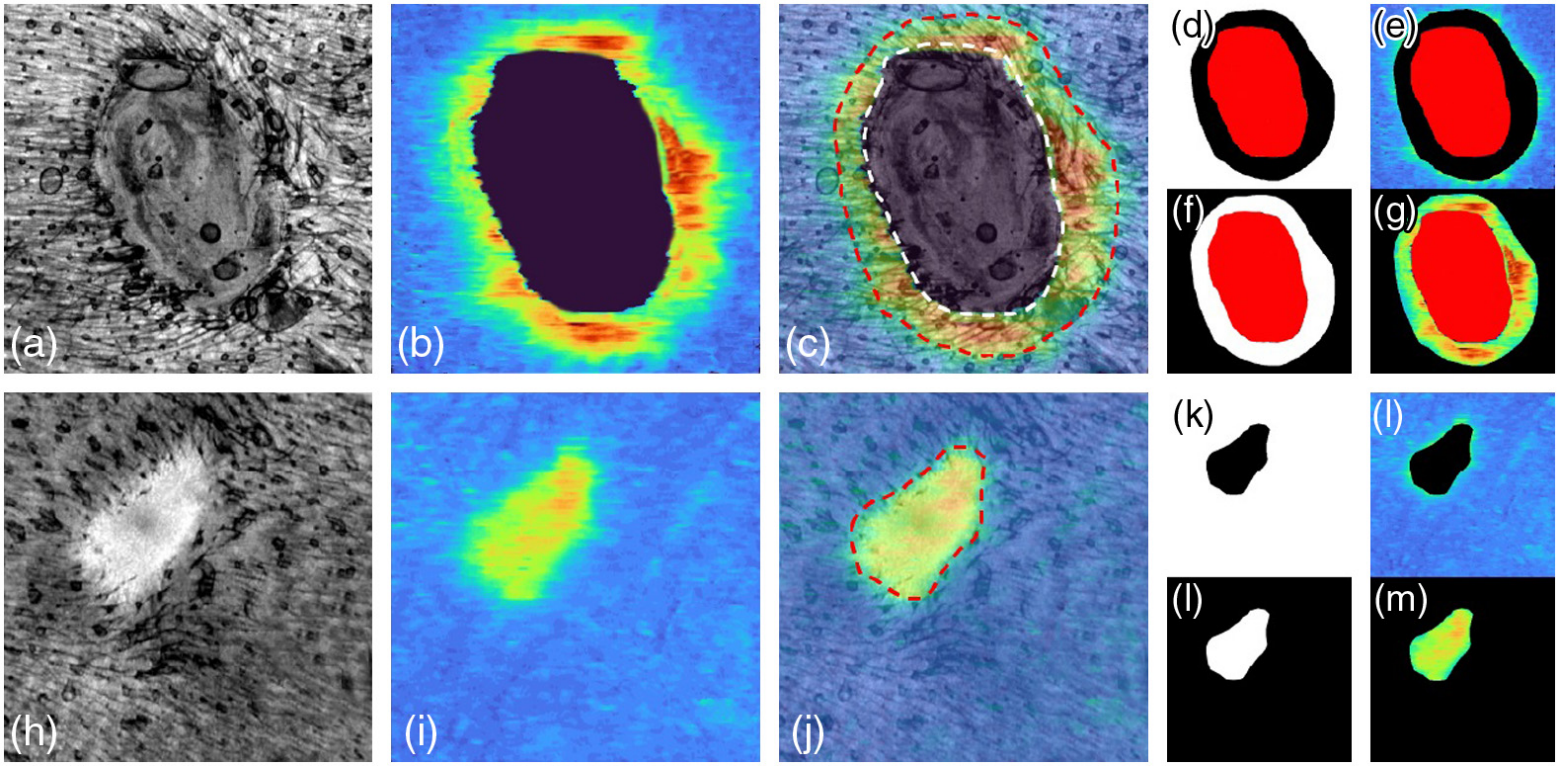
Mask preparation for quantitative the epidermal thickness during the wound healing. (a), (h) MIP *en face* projected structural images in dermis layer for day 3 (representative of not all the wound regions finish the re-epithelialization) and day 10 (representative of all the wound region has completed the re-epithelialization). (b), (i) The corresponding epidermal thickness maps of (a) and (h), respectively. (c), (j) The overlaid images of (a) and (b), (h) and (i), respectively. The red dashed line indicates the edge of wound area. The area inside the white dashed line indicates the region has not finished re-epithelialization. (d), (k) A negative mask derived from *en face* projected structure image of wound for day 3 and day 10, respectively. (e), (l) The multiplication of (c) and (d), (j) and (k), respectively. This allowed for quantification of epidermal thickness solely in healthy region. (k), (l) A positive mask derived from *en face* projected structure image of wound for day 3 and day 10, respectively. (g), (m) The multiplication of (c) and (f), (j) and (l), respectively. This allowed for quantification of epidermal thickness solely in the wound region. The red area in the day 3 is considered as remaining area to be re-epithelialization area which value is null.

The automated thickness measurement within different phases during wound healing was demonstrated in Fig. 5. Figures 5(a) and 5(h) show a representative *en face* structural image for wound healing day 3 and day 10, respectively. The corresponding *en face* epidermal thickness maps was shown in Figs. 5(b) and 5(i). A color code was subsequently applied to represent a thickness range of 0 to 131.25  μm (0 to 35 pixel). It was obtained by calculating the depth separation between the upper and lower boundaries of predicted segmentation result by deep-learning networks at each A-line. The overlays of *en face* thickness map and *en face* structure image in wound at day 3 and day 10 are shown in Figs. 5(c) and 5(j), respectively. In wound healing day 3, the region inside the white dashed line indicates area remaining to be re-epithelialization while the red dashed line highlights the edge of the wound. The area between the white and red dashed line is considered as newly generated epidermis region in the wound while the area outside the red dashed line is regarded as a healthy region. Since the full re-epithelialization was completed in day 10, the region inside red dashed line is a new epithelial region; meanwhile, it can also be considered as a wound area. Figures 5(d), 5(f), 5(k), and 5(l) show the representative positive and negative masks for region-specific epidermal thickness measurements for wounds at day 3 and day 10. Red mask represents the area remaining to re-epithelialization which value is null. Further, the mask is used to calculate its corresponding thickness map [see Figs. 5(e), 5(g), 5(l), and 5(m)], allowing for epidermal thickness changes to be measured solely within the wound itself, and/or within an equivalent control site. A more reliable normalized method is defined as below to assess the process of re-epithelialization during wound healing:

Re_Epinor=NE_wound¯Epi_health¯, (3)

where $\overset{¯}{\mathrm{NE}\_\text{wound}}$ represents the mean epidermal thickness in the region of new growth epidermis in wound region, while $\overset{¯}{\mathrm{Epi}\_\text{health}}$ represents the mean epidermal thickness in adjacent healthy region. Re_Epinor is epidermal thickness in the wound area normalized by its surrounding area. Since the scab region is always above the wound, it can be easily demonstrated by calculating the distance between the upper and lower boundaries of predicted red mask at each A-line.

The normalized epidermis and scab thickness were averaged and represented as a mean value ± standard error of the mean. Groups were compared with non-paired t-tests in the adjacent phase. In order to indicate statistical meaning, P-value below 0.05 was indicated. Four levels are shown in the graphs: *, P-value<0.05; **, P-value<0.01; and ***, P-value<0.001.

## 3 Results

Two experts examined the OCT frames from two mice and selected random 2000 B-scan images that corresponded to the ROI. Afterward, the contours of the epidermis and scab layers were marked independently. The thickness of both layers is predicted by the deep-learning algorithms and by the experts for comparison.

### 3.1 Qualitive Segmentation Accuracy Analysis Based on CNN Deep-Learning Networks

**Fig. 6:**
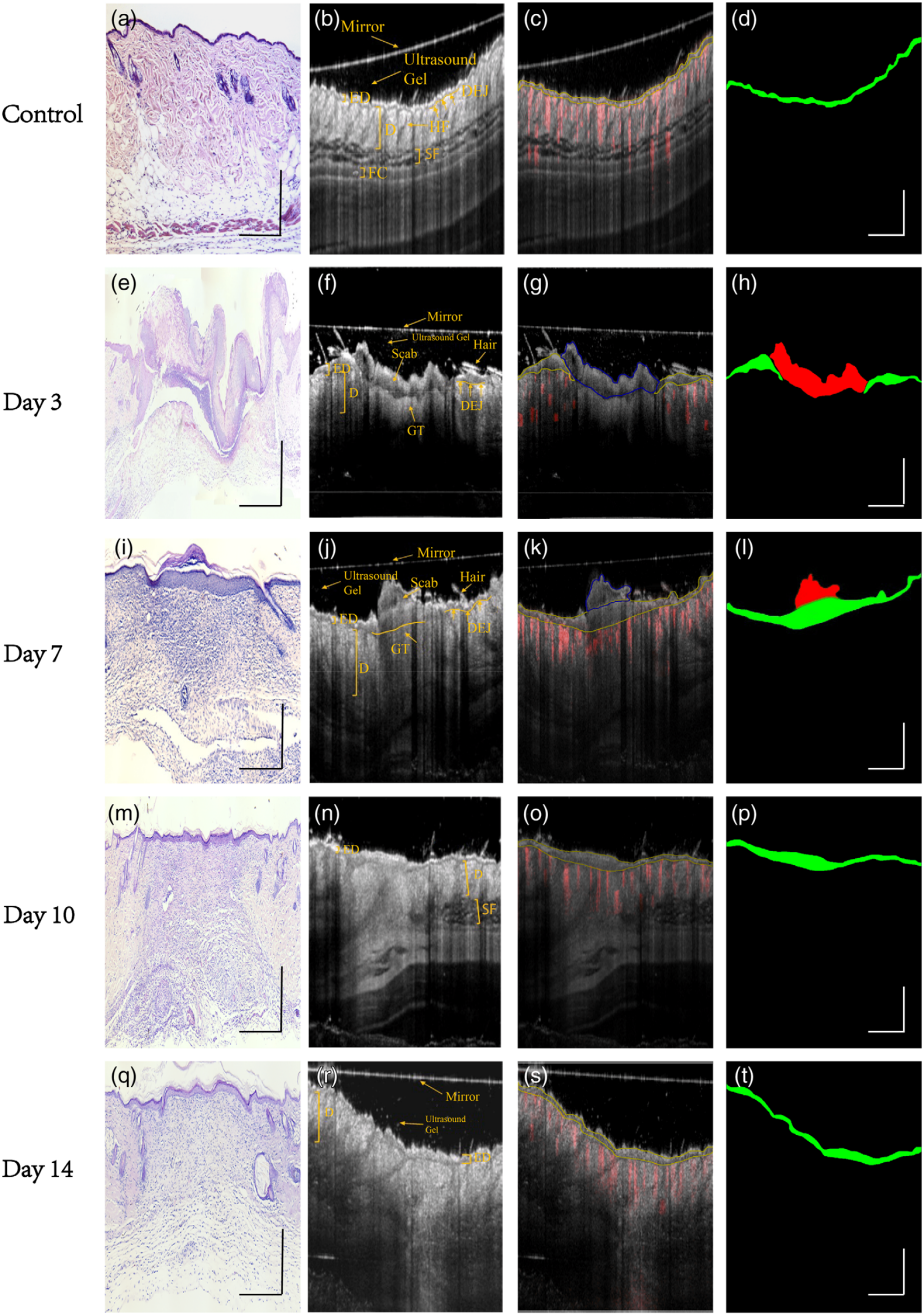
Experts’ manual segmentation strategy. (a), (e), (i), (m), and (q) Representative histology images (H&E staining) for control, wound healing day 3, day 7, day 10, and day 14, respectively. (b), (f), (j), (n), and (r) Cross-section OCT images and structure annotation of corresponding histology images of (a), (e), (i), (m), and (q). (c), (g), (k), (o), and (s) Overlay of cross-section structure images and its corresponding cross-sectional B-frames of the vasculature during normal and healing states. Blue contour represents scar area while yellow contour highlights the epidermis region which is marked by experts. (d), (h), (l), (p), and (t) Automatically generated mask according to the manual segmentation contour where the red mask represents scab region while green mask represents epidermis region, black color represents remaining area including other anatomy structure and background information. HF, hair follicle; ED, epidermis; D, dermis; GT, granulation tissue; DEJ, dermal-epidermal junction; SF, subcutaneous fat; and FS, fascia. Scale bar = 1 mm.

Qualities segmentation results from experts are shown in Fig. 6. The histology images shown in Figs. 6(a), 6(e), 6(i), 6(m), and 6(q) were used to confirm and validate the position of essential layer information in corresponding OCT imaging. According to the research of Israelsen et al.^47^ and the aid of corresponding histology images, the first clue to identify the epithelial in OCT images can be found during the wound healing, which is usually based upon recognizing the low-signal band region due to its lower scattering effect [see Figs. 6(b), 6(f), 6(j), 6(n), and 6(r)]. In the control data, the boundary between ED and D has high contrast and the epidermis appears as a thin and flatten layer. However, in the wound region, the contrast between the epithelial and subepithelial zone was prominently reduced and it highly degraded the visualization of the DEJ layer. Thus the epidermis region in the wound area can indirectly be defined accurately via microvascular information, as it typically coincides with the onset of re-epithelialization.^18^ Cross-sectional OCTA signal (showed as red signal) overlaid on its corresponding OCT images to assist finding the position of epidermis region, which can be illustrated as yellow contours in Figs. 6(c), 6(g), 6(k), 6(o), and 6(s). The scab above the wound consists mainly of necrotic tissue and presents poor scattering and highly surface reflection in OCT datasets. The blue contour in Figs. 6(c), 6(g), 6(k), 6(o), and 6(s) highlights the scab region. Figures 6(d), 6(h), 6(l), 6(p), and 6(t) show the automatic generated mask according to the manual segmentation contour which is the human-annotated ground truth of scab and epidermis region. The red-colored mask represents scab region while green colored mask defines the epidermis region. The black area means other structures including background.

**Fig. 7:**
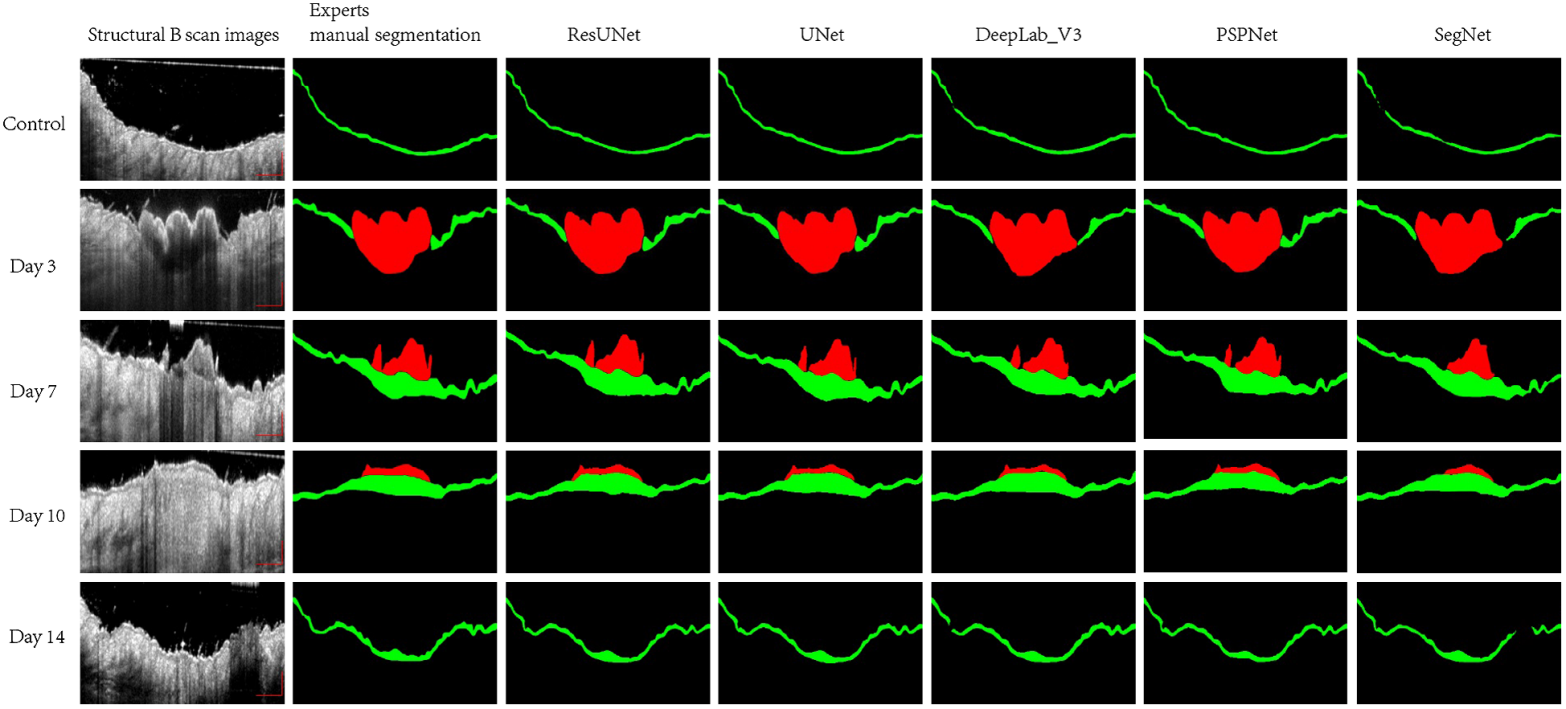
Segmentation results of representative OCT B-scan images in control, day 3, day 7, day 10, and day 14 postinjured. First column: the magnified representative cross-section B-scan images from validation datasets in control, day 3, day 7, day 10, and day 14 postinjured. Segmentation results with expert annotations (second column) and with segmentations by Res-UNet, U-Net, DeepLab-V3, PSP-Net, and Seg-Net (third to seventh columns, respectively). Scale bar represents 500  μm.

The qualitative comparison of segmentation results of representative 2D B-scan OCT images in the validation dataset is presented qualitatively in Fig. 7. The first column is the original magnified B-scan images; the second column is corresponding annotations from the first expert; the third to seven columns are the predicted results by five different CNN-based models (Res-Unet, U-Net, DeepLab-V3, PSP-Net, and Seg-Net model), respectively.

First row in Fig. 7 shows the result of epidermis segmentation in control data, all of the five models showed an acceptable result as a thin layer along the skin surface. However, compared with Res-UNet, U-Net, and PSP-Net model, the connectivity of epidermis prediction is worse in DeepLab-V3 and Seg-Net model. Although there is high visual agreement between segmentation results in day 3 of five different models with expert segmentation (shown in the second row of Fig. 7), the DeepLab-V3 and Seg-Net model have a problem in recognizing the thickened epidermis closed to the edge of scab. As demonstrated in fifth row of Fig. 7, when there is shadow artifact involved, the Seg-Net model fails to segment epidermis in this region. Other models are more robust to the low SNR and shadow region caused by hyper-reflection or hair.

### 3.2 Quantitative Segmentation Model Comparison

In order to compare the performance of deep-learning-based segmentation approach using five different models, seven different metrics are employed for a quantitative assessment of segmentation accuracy including precision (p), recall (r), F1-score (F1), F2-score (F2), intersection over union (IoU), Dice similarity coefficient (DSC), and average symmetric surface distance (ASSD). Definition of all seven quantitative parameters of segmentation accuracy can be found in the Appendix.

**Fig. 8:**
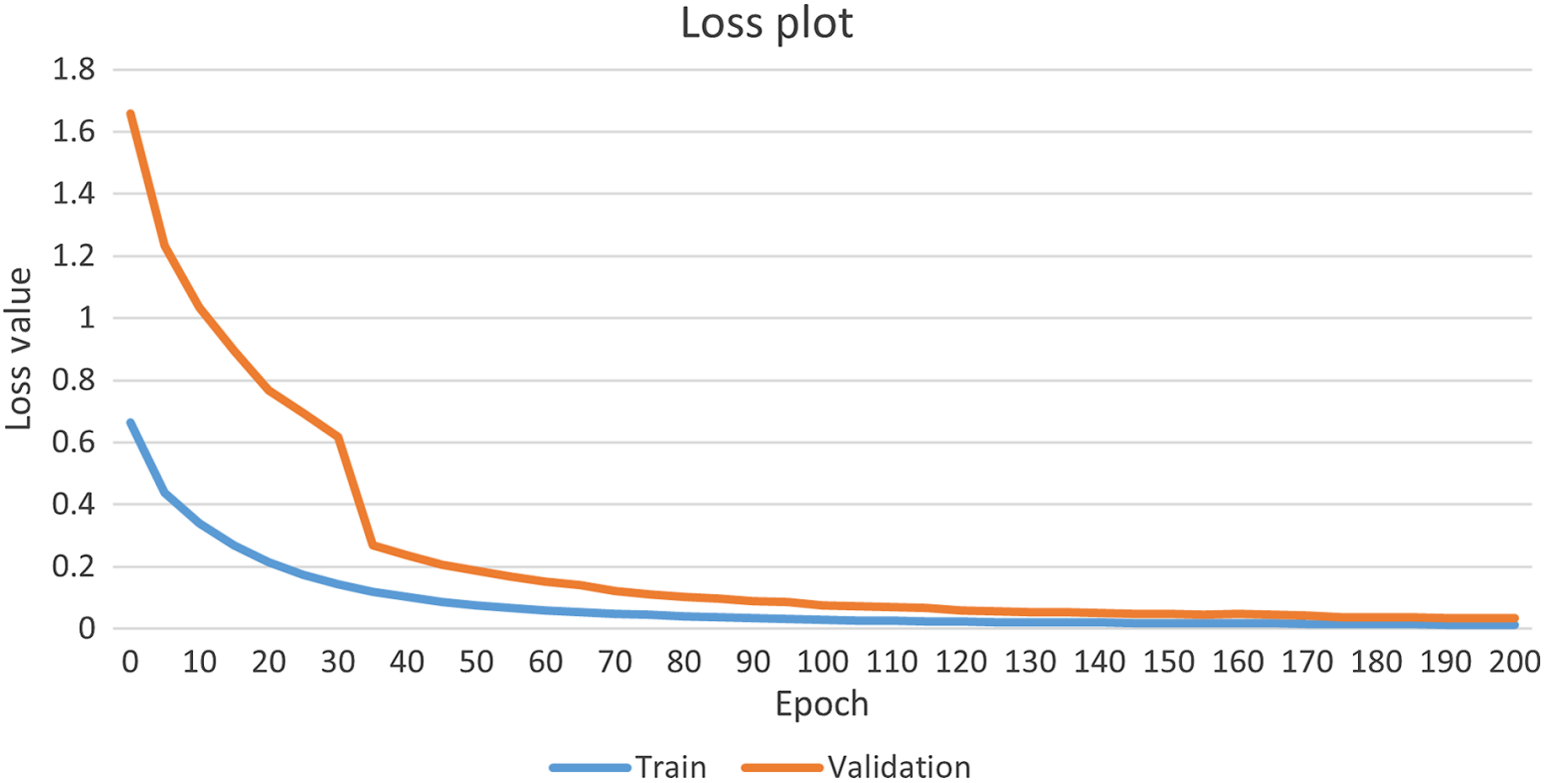
Training loss and validation loss of U-Net model.

As shown in Table 2, DeepLab-V3 model has the highest mean recall value (0.923); however, it suffers from a poorer precision value with 0.857 compared with U-Net. DeepLab-V3 in some cases was not able to find intact epidermis and scab region. A similar trend is observed in the quantitative results of Seg-Net, which has a high recall but low precision. The results for the recall and precision are similar across Res-UNet, U-Net, and PSP-Net models cross all the wound recovery timeline. Notably, the mean IOU, F1_score, DSC, and ASSD of U-Net model outperformed other models. Meanwhile, the U-Net model has a good compromise for the recall and precision values, which can further confirm that the U-Net can effectively predict the epidermis pixels. Furthermore, the ASSD based on U-Net model has the smallest variation with a range of 6.832  μm of mean absolute error. Table 3 compares the computational cost and calculation speeds for prediction of one cross sectional image with size of 512×512. The result reveals that U-Net model runs considerably faster than other CNN-based architecture with 6.7 ms per images and achieves the moderate computation complexity. Size of training parameters in DeepLab-V3 is lowest but achieve much slower speed during testing. Our quantitative results demonstrated that the best performance of five deep-learning models is the U-Net model. Additionally, Fig. 8 shows that both training loss and evaluation loss decrease at around 40 epochs and both smoothly down to the same level. It shows that the model does not overfit the training data.

**Table 2:** Mean evaluation metric (IOU, recall, precision, F1_score, F2_score, DSC, and ASSD) with standard deviation of validation data (bold font highlights the best indicator).

	Mean IOU	Recall	Precision	F1_Score	F2_Score	DSC	ASSD (μm)
Seg-Net	0.826 ± 0.072	0.901 ± 0.052	0.82 0 ± 0.04 2	0.862 ± 0.044	0.885 ± 0.047	0.905 ± 0.067	28.59 ± 10.29
Res-UNet	0.859 ± 0.053	0.889 ± 0.028	0.884 ± 0.032	0.886 ± 0.024	0.888 ± 0.036	0.924 ± 0.046	20.28 ± 8.49
U-Net	**0.875 ± 0.033**	0.906 ± 0.043	0.882 ± 0.027	**0.894 ± 0.036**	0.901 ± 0.032	**0.933 ± 0.033**	**18.28 ± 6.83**
PSP-Net	0.859 ± 0.046	0.880 ± 0.023	**0.892 ± 0.038**	0.886 ± 0.057	0.882 ± 0.042	0.884 ± 0.033	20.16 ± 9.46
DeepLab-V3	0.834 ± 0.058	**0.923 ± 0.062**	0.857 ± 0.034	0.889 ± 0.053	**0.909 ± 0.055**	0.909 ± 0.057	22.58 ± 8.05

**Table 3:** Comparison of computational complexity and calculation speed for five CNN-based model.

	Seg-Net	U-Net	Res-UNet	PSP-Net	DeepLabV3
Computation complexity (MB)	112.33	69.25	124.28	204.40	43.68
Calculation speed (s)	0.0090	0.0067	0.016	0.015	0.026

### 3.3 Qualitative Result of Epidermis and Scab Thickness Map

**Fig. 9:**
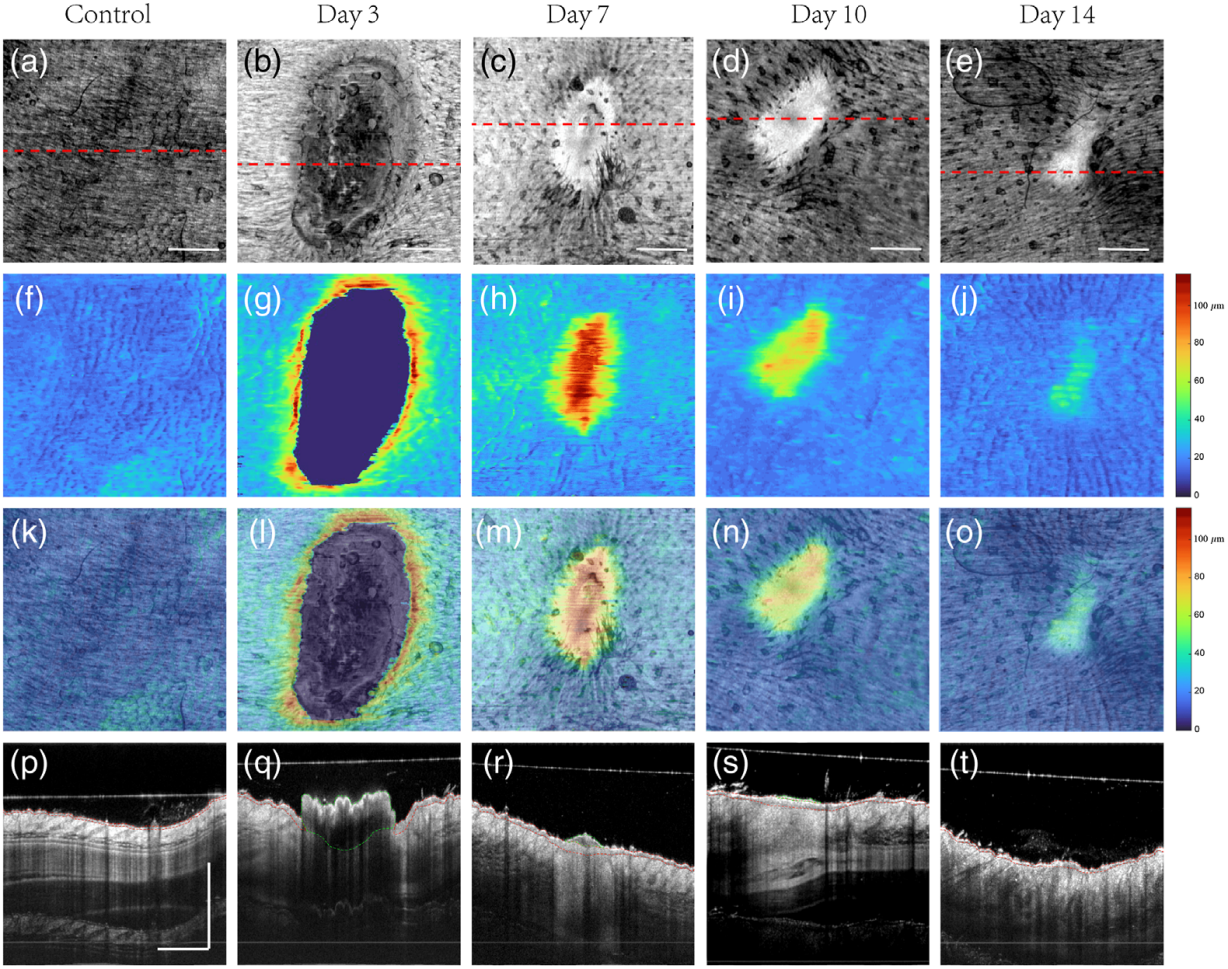
*En face* projected whole volume scan presenting the structure and epidermal features for normal and wound skin within four different phases. (a)–(e) MIP *en face* projected structure images during normal and different healing states (day 3, day 7, day 10, and day 14 postinjury). The red dotted line on each *en face* image indicates where the corresponding cross-sectional B scan images from mouse were taken from. (f)–(j) MIP *en face* epidermal thickness maps by U-Net deep-learning network. The range of the color bar on the right side is 0 to 131.25  μm (0 to 35 pixel). (k)–(o) Overlay of *en face* projected structure images and its corresponding epidermal thickness maps. (p)–(t) Cross-sectional B frames of structure during normal and healing states. The area inside the red dashed line represents the epidermis region while the area inside the green dashed line represents the scab obtained by deep-learning network. Scale bar represents 1 mm.

Figures 9(p)–9(t) show the selected cross-sectional B-scan images corresponding to the dashed red lines in Figs. 9(a)–9(e), respectively. The *en face* projection of adjacent normal skin surrounding the wound [see Fig. 9(a)] typically gives homogenous distribution of the texture. The *en face* epidermal thickness maps [Fig. 9(f)], together with the corresponding cross-sectional B-frame of normal skin, which demonstrate the epidermis in normal mouse skin is a flat, homogeneous, intact, and thin layer.

From red dashed line circled in Fig. 9(q), we observed that the thick scab was forming to cover the wound area in day 3 postinjury. The formation of granulation tissue was mainly situated at the bottom of the scab, and it already fully filled the wound bed. Thickened epidermis is observed at its cut margins, in order to recruit keratinocytes, but they have not bridged the whole incision in this stage. The epidermal thickness map generated by our proposed deep-learning network is illustrated in Fig. 9(g), together with overlapped images with *en face* structure image [Fig. 9(l)], which again confirm the thickened epidermis is located in the peripheral of wound area.

In the healing period 7-days after surgery, the re-epithelialization grows rapidly, and the scab is replaced gradually which can be shown from Figs. 9(c) and 9(r). The incisions were completely bridged with multi-layers of newly synthesized epithelial cells. The progressive increase in its collagen fibers and fibroblasts were placed in granulation tissue (see bright region in the wound). As shown in Figs. 9(h) and 9(m), the thickened region is well correlated with corresponding wound region of *en face* structure image. The epidermis gradually becomes thicker when it reaches to the center of the wound.

The observation from Figs. 9(d) and 9(e) and its corresponding cross-section image [Figs. 9(a) and 9(t)] in day 10 and day 14 revealed similar structural information as the major components in granulation tissue is extracellular matrix (ECM) and there is little scab remaining on the top of the wound region. During wound contraction, re-epithelialization showed a lower number of epithelial layers in wound healing day 10, while in day 14, its thickness was similar to intact epidermis. The finding can be confirmed again by the obtained epidermal thickness mapping result (shown in Figs. 9(i) and 9(j)]. According to the overlaid region in Figs. 9(n) and 9(o), it revealed thinner epidermis layer compared to that in day 7 postinjury when the process of epithelial contraction just started.

### 3.4 Quantitative Result of Epidermis and Scab Thickness Map

**Fig. 10:**
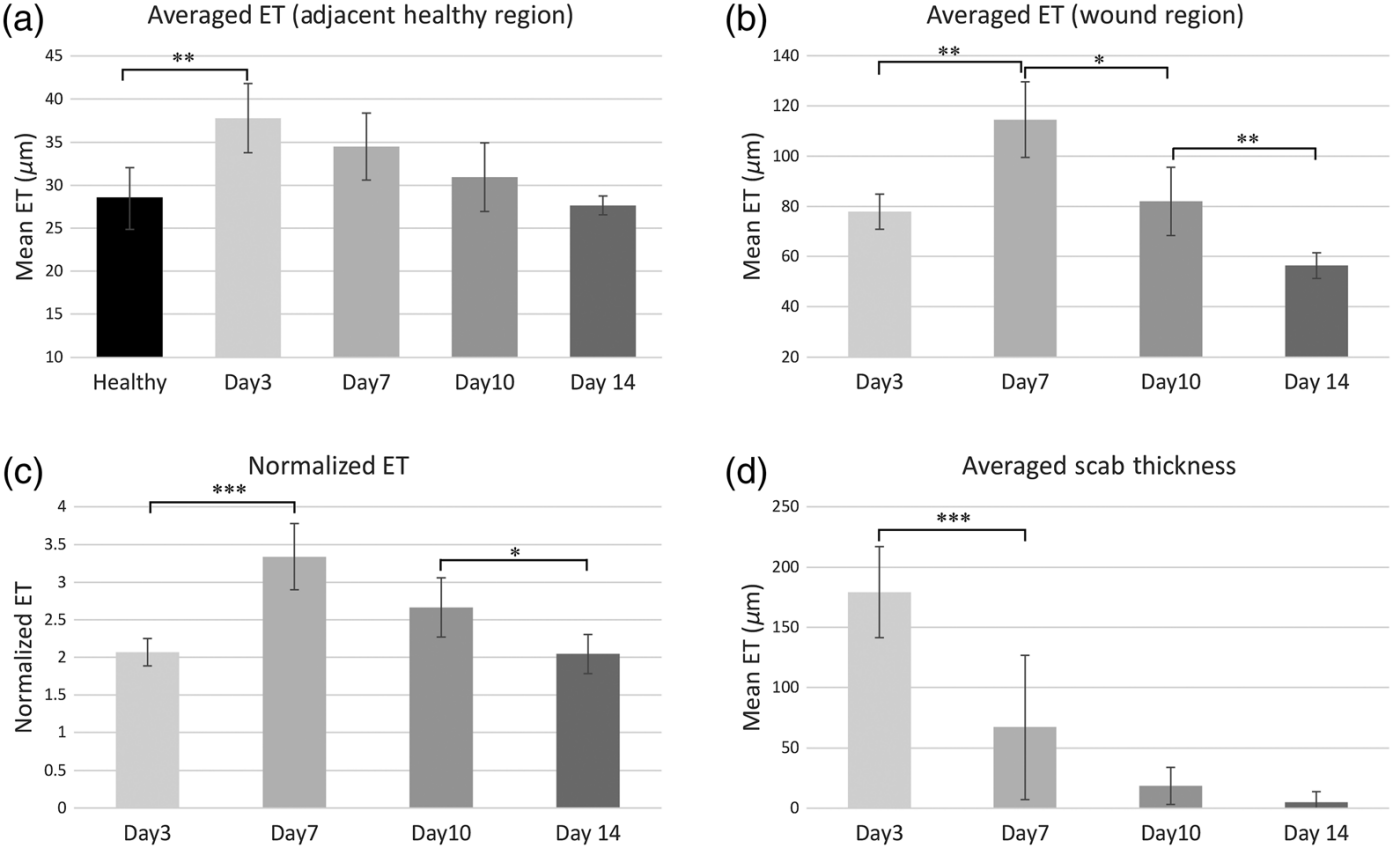
Quantitative result of epidermis and scab thickness spanning the whole healing process. (a) Mean epidermis (ET) thickness in surrounding healthy region; (b) the mean thickness of newly generated epidermis in wound region; (c) the mean normalized epidermal thickness; and (d) the mean thickness of scab region. Error bars represent the standard deviation. *Represents p-value<0.05, **represents p-value<0.01, ***represents p value<0.001, ET, epidermal thickness.

The selected U-Net model was applied to predict the epidermal and scab region of test datasets which consisted of 36 OCT volumes (totally 14,400 OCT images). Figure 10 provides the quantification results of epidermal thickness and scab thickness parameters at the days 3, 7, 10, and 14 postinjuries, alongside its corresponding results from normal skin region. Figure 10(a) shows the averaged epidermal thickness taken solely from the healthy region (0.4  cm×0.4  cm) surrounding the wound. The epidermal thickness is showed significant difference between control and day 3 postinjury (P-value=0.0082), which indicates that the inflammation at day 3 thickens the epidermis surrounding the wound area. Additionally, although there was no statistically significant difference of averaged epidermal thickness in the healthy region between different stages of wound healing, it shows a decreased trend which demonstrates that the inflammation subsides gradually back to normal. Figure 10(b) shows the averaged epidermal thickness taken solely from the wound area. The keratinocytes start to be recruited at the edge of the wound to get a thicker epithelial (77.8±7.0  μm) by day 3. Day 7 postinjury is a unique time when all wound regions have completed epithelialization and the mean epidermal thickness in the wound has reached its peak (114.6±15.0  μm) before gradually decreasing as wound healing progresses on day 10 (82.0±13.6  μm) and day 14 (56.3±5.1  μm). The epidermal thickness between all the successive stage showed significant difference (day 3 to day 7: P-value=0.0028, day 7 to day 10: P-value=0.013, day 10 to day 14: P-value=0.0018). In order to measure the change of re-epithelialization in response to adjacent healing part, the statistical analysis of normalized epidermal thickness is present in Fig. 10(c). The trend was similar to that in Fig. 10(b). However, no significant difference can be shown between day 7 and day 10 postinjury. Shown in Fig. 10(d) are the averaged scab thickness measurements at the days 3, 7, 10, and 14 postinjuries. Although mean scab thickness decreased across different stages, the variance among individuals is massive. The significant difference is only visualized between day 3 and day 7, in which P value is smaller than 0.0005.

## 4 Discussion

The concept of automated segmentation using deep-learning method has become increasingly popular in OCT imaging; however, comparatively little has been applied in dermatology and the validation of proposed models were limited to healthy samples. The proposed pipeline in this study offers innovative and efficient way to segment the epidermis and scab precisely. According to the segmentation output, *en face* epidermal and scab thickness maps as well as its quantitative parameters are obtained automatically spanning the whole healing process.

Image preprocessing approach, including multi-volume registration and autocropped algorithm, was applied to reduce the movement and speckle noise as well as enhanced the training efficiency. A standard and systematic manual segmentation method for mouse model is validated by its corresponding histology images and angiograms via OCTA technique. Five different architectures of CNN-based segmentation models (Res-UNet, U-Net, DeepLab-V3, PSP-Net, and Seg-Net) were applied for comparison. All the model trained with 200 epochs without overfitting. All the five models are able to identify basic expressive features of epidermis and scab. In comparison to Seg-Net and DeepLab-V3 model, the U-Net, Res-UNet, and PSP-Net models offer more robust segmentation result against the shadow noise, hyper-reflection and low SNR region, resulting in better connectivity in the segmented epidermis structure. DeepLab-V3 and Seg-Net models also present a higher recall and poorer precision value compared with other three models. Furthermore, U-Net and Res-UNet were more adaptive to different stages of wound healing which can recognize various thickened epidermis, epithelial tongue (mostly in day 3), and scab region. U-Net model showed both higher DSC scores and lower ASSD compared with Res-UNet which means it improves the segmentation accuracy in the area of object contours. Additionally, U-Net shows an excellent balance between precision and recall which agrees with the highest F1-score (Table 2). According to the quantitative assessment, the U-Net model is then selected to predict the new OCT datasets in test data (∼3  s to predict one data volume) which requires no additional human input.

By analyzing the *en face* epidermis and scab thickness maps quantitatively with its corresponding *en face* structural images in this study, we were able to formulate an approximate healing timeline schematic for full-thickness incision wound in healthy mouse. It includes inflammation, proliferation, and remodeling phases, which are not strictly separated from each other, and their processes freely blend together.^1^ Control data were taken from the adjacent healthy region of the wound and its epidermal thickness maps is homogeneous. Based on our model, the mean epidermal thickness in back site of the healthy mice model is 28.0±3.59  μm, which is slightly thicker than another research gives 21.9±4.29  μm by measuring 15 to 20 random site based on histology images.^48^ The difference is taken into account for *in vivo* OCT scanning, which has a lower effect on tissue dehydration. Based on epidermis thickness maps and its corresponding cross-section structure images, it demonstrates the day 3 postinjured is in the overlapped phase of late inflammatory and early proliferation. Loosen granulation tissue tends to fill out the wound bed, which can be regarded as the first step of the proliferation stage. Together with the protection of thick scab (179.3±37.9  μm) is forming above the wound to rebuild the damaged area. In all the cases at day 3 postinjured, the epithelial does not fully bridge the wound and its thickness in the re-epithelialization region was 2.07±0.18 times thicker than the healthy region due to mitotic activity and proliferation of nearby basal cells. The wound healing at day 7 and day 10 was characterized by almost total regression of the inflammatory process as the new epithelial were completely bridged the wound bed with newly synthesized epithelial cells. Re-epithelialization showed a higher number of newly formed epithelial layers 3 to 4 times thicker than the surrounding healthy region in day 7 postinjured. By day 10, the newly epidermis formation is only ∼2.5 thicker than the healthy region, thinner than day 7, indicating that the process of epidermal contraction has begun. Moreover, the bright tissue surrounding the contracting wound is thought to be a feature of new ECM (mainly composed by collagen fibers),^49^ which at this stage serves two purposes: to provide structural strength and to facilitate the migration of various cell types responsible for wound healing.^50^^,^^51^ Meanwhile, the scab is gradually rejected. In the wound healing day 14, the thickness of epidermis in the wound was reduced to approximately two times thicker than the normal area. In this stage, the wound is contracting, and the bright area considered as ECM region become fade, which reflects the collagen type III being converted to type I. Additionally, almost all the scab is replaced by the new tissue after wound healing day 14. At day 14, re-epithelialization process finishes as the inflammatory signs disappear.

Due to the superior segmentation accuracy and speed outlined in this study, our algorithm is able to provide the basis for quantitative assessment of the wound based on its state of re-epithelialization. Moreover, the normalized epidermal thickness can be serving as an essential hallmark to describe the normal re-epithelialization process of the mouse model. According to the deviations from normal epidermis contracting speed in each timeline, it is promising to extent the research in assessing the drug efficacy or physical factors (laser light and magnetic field) by which the wound healing can be favorably influenced.

It should be acknowledged, however, that this study has some limitations. First, due to limited penetration depth in OCT, it is difficult to observe keratinocytes that have migrated beneath the thick scab (commonly happened on day 3 postinjury). Secondly, the study comprised only two mice (six sites for each stage) of the full-thickness wound. In the future, the algorithm should be further validated by including wounds of different severities within a variety of scenarios. It is also worth noting that the images used in the current study are from healthy mouse, and therefore further work is required to examine the proposed segmentation methods in cases of different disorders, such as a chronic wound. Additionally, from a technical perspective, the future lines of work will focus on adapting the network architecture to other models, especially for human. This can be performed by doing transfer learning,^52^^,^^53^ where the model is fine-tuned with data of the target domain with only a small number of annotated images, which will enable the proposed method to achieve clinical utility. Additionally, a novel technique has been developed recently named zero-shot learning^54^^,^^55^ which can predict a class that was omitted from a training set. Using this technique, it is promising to be saving more time for the training of new datasets with different conditions of the model.

## 5 Conclusion

We present a novel pipeline that automatically detects epidermis and scab layer using deep-learning frameworks. The method is validated by comparing the algorithm-obtained segmentation results with the golden-standard method (manual segmentations from human experts). Our proposed deep-learning method shows promising results in segmentation accuracy and automated quantification of epidermal and scab thickness of mouse skin data within the standard healing timeline. This pipeline is more efficient than manual labeling and makes OCT useful in the clinical and research arenas. Furthermore, the automatic segmentation and thickness measurements of data within different phases of wound healing demonstrates that our system provides a robust, quantitative, and accurate method for serving as a standard model for further research into pharmacological and physical factors.

## 6 Appendix: Evaluation Metrics

• Precision (p), recall (r), F1 score, and F2 score: These measures evaluate the fraction of correctly predicted instances of the validation datasets. Given a number of true instances GT and number of predicted instances Pred by a method, precision is the fraction of predicted instances that were correctly found:
  $$p=\frac{\mathrm{TP}}{\text{Pred}},$$ (4)
  where TP denotes number of true positives and recall is the fraction of ground-truth instances that were correctly predicted:
  $$r=\frac{\mathrm{TP}}{\mathrm{GT}}.$$ (5)
  Ideally, the best methods should have jointly high precision and recall. F1 and F2 scores give a single score to capture this desirability through a weighted β harmonic means of precision and recall:
  $$F\beta =(1+\beta^{2})*\frac{p*r}{(\beta^{2}*p)+r}.$$ (6)
  β equal to 1 represents the F1 score while β equal to 2 represents F2 score.
• IoU can be defined as follows:
  $$\mathrm{IoU}(\mathrm{GT},S)=\frac{\left|\mathrm{GT}\cap \mathrm{Pred}\right|}{\left|\mathrm{GT}\cup \mathrm{Pred}\right|},$$ (7)
  where |·| denote the set cardinality. The IoU is 0 for no overlap and 1 for perfect overlap.
• DSC is a spatial overlap measure for segmentation which is similar to IoU. It can be defined as
  $$\mathrm{DSC}(\mathrm{GT},S)=\frac{\left|\mathrm{GT}\cap \mathrm{Pred}\right|}{\left|\mathrm{GT}|+|\mathrm{Pred}\right|}.$$ (8)
  DSC is 0 for no overlap and 1 for perfect overlap. It is related to IoU:
  $$\mathrm{DSC}=\frac{2*\mathrm{IoU}}{1+\mathrm{IoU}}.$$ (9)
• ASSD: The size of the segmented areas has an effect on the DSC, since misclassifications have a stronger impact on smaller areas than on larger ones. Therefore, we additionally use ASSD in this work. Let NS={p0,…,pn1} and NGT={q0,…,qn2) be a subsets of a predicted segmentation S and a ground truth GT with NS⊆Pred and NGT⊆GT containing surface points. The surface distance SD between SP and SG is then defined as
  $$\mathrm{SD}(S_{p},S_{G})=\overset{n2}{\underset{i=0}{\sum }}\min{\left\|p_{j}-q_{i}\right\|}_{2}.$$ (10)
  The surface distance can then be used to determine the ASSD:
  $$\mathrm{ASSD}=\frac{\mathrm{SD}(S_{P},S_{G})}{2n_{2}}+\frac{\mathrm{SD}(S_{P},S_{G})}{2n_{1}}.$$ (11)
